# Five Minutes’ Value

**Published:** 1864-04

**Authors:** 


**Five Minutes' Value.**—A number of years ago, it was a custom of the orthodox churches in Boston to furnish about a dozen teachers, who would voluntarily go to the prison on Sabbath forenoon, to instruct classes of the convicts in a Sabbath-school in the chapel.

Hon. Samuel Hubbard was one cf those who went. Near the close of the time devoted to instruction, the chaplain said:

"We have fine minutes to spare. Mr. Hubbard, will you please to make a few remarks?"

He arose in a calm, dignified manner, and looking at the prisoners, said:

"I am told that we have five minutes to spare. Much may be done in five minutes. In five minutes, Judas betrayed his Master, and went to his own place. In five minutes, the thief on the cross repented, and went with the Saviour to Paradise. No doubt many of those before me did that act in five minutes which brought them to this place. In five minutes, you may repent, and go to Paradise—or will you imitate Judas, and go to the place where he is? My five minutes have expired."

